# Super-Enhancer Induced IL-20RA Promotes Proliferation/Metastasis and Immune Evasion in Colorectal Cancer

**DOI:** 10.3389/fonc.2021.724655

**Published:** 2021-07-15

**Authors:** Dingye Yu, Xiao Yang, Jianwei Lin, Zichao Cao, Chenghao Lu, Zheyu Yang, Minhua Zheng, Ruijun Pan, Wei Cai

**Affiliations:** ^1^ Department of General Surgery, Ruijin Hospital, Shanghai Jiaotong University School of Medicine, Shanghai, China; ^2^ Shanghai Minimally Invasive Surgery Center, Ruijin Hospital, Shanghai Jiaotong University School of Medicine, Shanghai, China

**Keywords:** IL-20RA, colorectal cancer, super-enhancer, tumor progression, immune evasion

## Abstract

Unveiling key oncogenic events in malignancies is the key to improving the prognosis and therapeutic outcome of malignancies. Lines of evidence have shown that super-enhancers control the expression of genes that determine the cell fate, but the oncogenic super-enhancers in colorectal cancer (CRC) and their impact on carcinogens remain largely unexplored. Here, we identified a new oncogenic super-enhancer-regulated gene, IL-20RA, in CRC. Using the integrative analysis of H3K27ac ChIP-seq and RNA-seq in CRC tumors and normal colon tissues, we obtained a series of oncogenic super-enhancers in CRC. We found that super-enhancer inhibition by JQ-1 or iBET-151 suppressed the growth of tumor cells and inhibited the expression of IL-20RA. We found that IL-20RA was highly expressed in the tumor tissue of CRC and related to the advanced stage. Further functional studies showed that knockdown of IL-20RA inhibited the growth and metastasis of CRC. In addition, we found that IL-20RA was involved in regulating oncogenic and immune pathways and affecting the expression of genes related to cell proliferation and immune evasion in CRC. Together, our study demonstrated a novel oncogene in CRC and shed new light on oncogenic super-enhancer contributions to cell proliferation and immune escape.

## Introduction

Colorectal cancer (CRC) was the third most commonly diagnosed cancer and the second leading cause of cancer death in global cancer statistics in 2020 (1). In China, the mortality of CRC increased from 6 (1990) to 13 (2017) per 100,000 individuals (2). Radical resection is recognized as the main treatment for CRC. However, most CRC patients are diagnosed at an advanced stage or with metastasis, and require chemotherapy, immunotherapy, or targeted therapy. Therefore, finding novel targets at the molecular level is critical to improve the early diagnosis, treatment and prognosis of CRC.

Taking advantage of high-throughput sequencing, many genes differentially expressed between CRC and normal colorectal tissues have been identified, but which genes might play essential roles during the oncogenesis of CRC remains elusive. Transcription deregulation is one of the hallmarks of cancers. Transcription deregulation leads to abnormal expression of genes related to carcinogenesis, such as oncogenes and tumor suppressor genes (3, 4). In recent years, studies on transcription deregulation have demonstrated that super-enhancers (SEs) are key regulators of essential genes in tumor cells (5). SEs are a large cluster composed of multiple enhancers with strong transcriptional activity, including enrichment of a high density of H3K27ac histone modifications and engaged binding of key transcription factors and cofactors (5, 6). It has been evident that chromosomal rearrangements reorganize SEs to oncogenes, which is called enhancer hijacking, resulting in increased expression of IGF2 in tumor cells (7). Nevertheless, essential super-enhancers in CRC remain largely unexplored, and an integrative analysis of CRC super-enhancers should locate some essential genes for the carcinogenesis of CRC.

Interleukin-20 receptor alpha (IL-20RA), located in chromosomal region 6q23, is a subunit of the IL-20RA/IL-20RB receptor dimer for IL-10 family members including IL-19, IL-20, IL-22, IL-24 and IL-26 (8). Previous studies have shown that IL-20RA is highly expressed in the skin, lung and reproductive organs targeted by the IL-10 family (9). IL-20RA expression was significantly increased in prostate cancer, breast cancer and non-small cell lung cancer (10, 11). It has been demonstrated that IL-20RA is correlated with inflammatory diseases and tissue repair, but the relationship between IL-20RA and CRC remains unclear.

Here, we conducted an oncogenic super-enhancer analysis in CRC and identified that IL-20RA was dysregulated by an oncogenic super-enhancer. We found that the expression of IL-20RA was significantly increased in tumor tissues compared with normal tissues. IL-20RA knockdown notably suppressed the proliferation, migration and invasion of colorectal cancer cells. IL-20RA regulated the expression of genes involved in the immune response, cell growth and angiogenesis. Thus, IL-20RA may serve as a novel potential target for colorectal cancer.

## Materials and Methods

### Identification of Super-Enhancers in Colorectal Cancer

H3K27ac ChIP-seq datasets were used to identify super-enhancers in CRC. Raw H3K27ac ChIP-seq data of primary CRC samples, normal colon tissues, and CRC cell lines were obtained from the GEO database (GSE). The fastq files were mapped to the human genome (hg38) using bowtie2 (12). Samtools (13) were used for sorting and indexing. The peaks were called using the Macs suite (version 1.4) (14). The super-enhancer was called using the ROSE algorithm (5, 15). The super-enhancer was annotated with the Homer suite (16).

### RNA-Seq Analysis

Raw reads were mapped to the reference genome (hg38) using STAR2 software (17). The read count matrix was obtained with the Ht-seq algorithm (18). For differential gene expression analysis, the DEseq2 package (19) was used. Genes with a two-fold change and FDR<0.01 were considered significantly changed. For the gene ontology analysis, the GOstat R package (20) was used.

The raw sequence data reported in this paper have been deposited in the Genome Sequence Archive (Genomics, Proteomics & Bioinformatics 2017) in National Genomics Data Center (Nucleic Acids Res 2021), China National Center for Bioinformation/Beijing Institute of Genomics, Chinese Academy of Sciences, under accession number HRA000982 that are publicly accessible at https://ngdc.cncb.ac.cn/gsa-human (21, 22).

### Ethics Statement

This study was approved by the Human Ethics Committee of Ruijin Hospital. Informed consent was obtained from all enrolled patients and healthy donors.

### Enrollment and Immunohistochemistry (IHC) Staining

A total of 118 colorectal cancer and paired adjacent normal tissues supplied by the Shanghai Minimally Invasive Surgery Center were used for analyzing IL-20RA expression. CRC staging was performed according to the UICC guidelines (8th Edition). All cases did not receive neoadjuvant chemotherapy. Tumor tissues and paired normal colorectal tissues which located more than 5 cm to the distal edge of the tumor were fixed with formaldehyde and embedded in paraffin. A tissue array was constructed for further immunohistochemistry (IHC) staining. The staining score of each tissue was calculated based on the widely used German semi-quantitative scoring system by three independent pathologists (23).

### Plasma and Enzyme-Linked Immunosorbent Assay (ELISA)

Plasma from 52 CRC cases and 17 healthy donors was used for the detection of IL-20RA. CRC cases were recruited from department of gastrointestinal surgery in Ruijin Hospital. They were diagnosed as single-site colorectal cancer for the first time and did not receive neoadjuvant chemotherapy. Healthy donors were recruited from medical center in Ruijin Hospital without any concomitant disease. The ages are 62.42 ± 12.04 for CRC cases and 52.32 ± 8.35 for healthy donors. ELISA assays were conducted with the IL-20RA ELISA Kit (RayBiotech, Atlanta, Georgia, USA) according to the manufacturer’s instructions.

### Cell Culture

CRC cell lines (LoVo, caco-2, HT29, SW620, SW480, HCT116, RKO) and HEK293T cells were purchased from American Type Culture Collection (ATCC, Manassas, VA) and cultured in F12K, L-15, RPMI 1640, or DMEM supplemented with 10% fetal bovine serum (FBS) and antibiotics (Gibco, Grand Island, NY, USA) under a humidified atmosphere containing 5% CO_2_ at 37°C.

### Cell Line Establishment

The sh-IL-20RA plasmid was generated using the pLVX-shRNA2 vector. sh-IL-20RA plasmids or vector and psPAX2 and pMD2.G were co-transfected into the HEK293T cells. The culture medium was collected after 48-72 hours and filtered for lentiviral transduction. Lentivirus carrying the empty plasmid was used as a control. The sequences specifically targeting IL-20RA silencing were: shIL-20RA#1: GCTATTCCATCTACCGATA and shIL-20RA#2: GCCCGCAAACGTTACAGTA.

### Cell Proliferation Assay

Cells were seeded in 96-well plates at 2000 cells per well and cultured in 100 μL of medium. Cell viabilities were determined at 0, 24, 48 and 72 hours by Cell Counting Kit 8 (Meilun, Dalian, China). The absorbance was measured at 450 nm by a microplate reader (BioTek, Winooski, VT, USA). The recombinant protein IL-20 (#13060-HNAE; SinoBiological, Beijing, China) was diluted to 20 and 100 ng/mL in medium.

### Cell Migration and Invasion Assay

For the migration assay, 1×10^5^ cells were seeded into the upper chambers of an 8-μM pore Transwell (Corning Life Science, MA, USA) in 200 μL of FBS-free medium. The lower chambers were filled with 600 μL of medium with 10% FBS. Cells on the topside of the filter were removed by scrubbing with swabs before measurement. The migration of cells to the lower side of the filter was determined by 0.1% crystal violet staining. The number of migrated cells was counted under a microscope. Each testing group contained at least three independent wells.

For the invasion assay, the topside of the filter was covered with Matrigel (Corning, NY, USA) diluted in medium at a 1:6 proportion. The follow-up experimental process was the same as the migration assay.

### Wound Healing Assay

LoVo cells were seeded into 12-well plates at more than 95% confluence and wounded with 200 μL pipette tips. The floating cells were removed using F-12K medium (Gibco, Grand Island, NY, USA) free of FBS and cultured at 37°C. Wound closure was photographed at 0, 24, and 48 hours.

### Quantitative Real-Time PCR

Total RNA from cultured cell lines was extracted using TRIzol reagent (Invitrogen, Carlsbad, CA, USA). Reverse transcription was conducted using qPCR RT Master Mix with gDNA Remover (Toyobo, Shanghai, China). Quantitative real-time PCR was performed using Hieff qPCR SYBR Green Master Mix (YEASEN Biotechnology, Shanghai, China) on an ICycler (ABI, California, USA, QuantStudio 12K Flex Software/v1.2.2). Fold expression was calculated using the 2-ΔΔCT method. Gene expression was normalized to GAPDH. IL-20RA (sense primer: 5’-3’ ACAAAGTGTTCCAAATGGGCT; anti-sense primer: 5’-3’ TGGGACCACGTTCTGTTTGAT) and GAPDH (sense primer: 5’-3’ GGACCTGACCTGCCGTCTAG; anti-sense primer: 5’-3’ GTAGCCCAGGATGCCCTTGA) were assessed.

### Western Blotting

The cell samples were lysed with RIPA buffer supplemented with PMSF at a ratio of 1:100. Equal amounts of protein samples were separated using a 10% SDS-PAGE gel and transferred onto PVDF membranes. Primary and secondary antibodies were used to bind the corresponding proteins. Primary antibodies included IL-20RA (mouse mAb, #sc-80065, 1:500; Santa Cruz, CA, USA), GAPDH (rabbit mAb, #5174, 1:5000; CST, Danvers, MA, USA), Snail (rabbit mAb, #3879, 1:1000; CST), and Slug (rabbit mAb, #9585, 1:1000; CST).

### Statistical Analysis

IBM SPSS Statistical software (version 22.0) was utilized for statistical analysis. Experimental data are presented as the mean ± SD. Significances between groups were examined using a two-tailed Student’s t test. The chi-square test or Fisher’s exact test was used for categorical variable comparison. P values < 0.05 were considered statistically significant.

## Results

### Identify 47 Super-Enhancer-Regulated Oncogenic Genes in Colorectal Cancer

**Figure 1:**
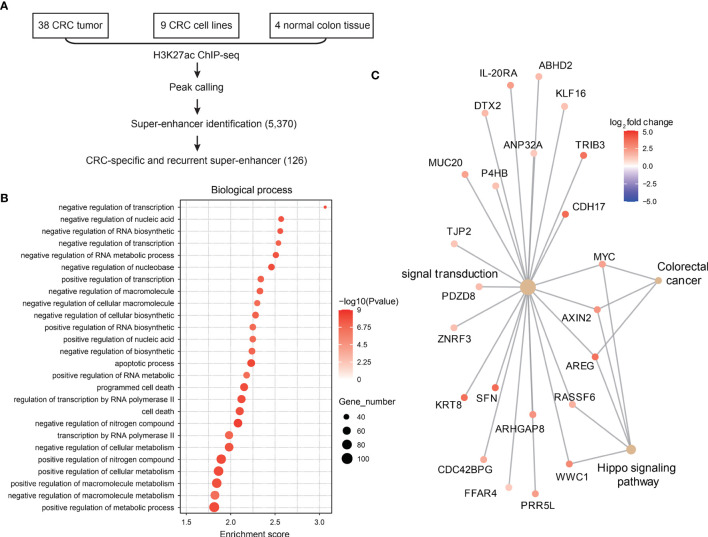
Landscape of super-enhancers in colorectal cancer. **(A)** Schematic illustration of super-enhancer-related oncogene identification in CRC. **(B)** Gene ontology analysis of genes related to CRC-specific super-enhancers. **(C)** Expression of CRC-specific oncogenic super-enhancer-related genes in CRC and normal adjacent tissues.

Since super-enhancers have been reported to regulate genes essential for cell identity and were shown to be critical regulators during carcinogenesis, we first explored CRC-related super-enhancers using H3K27ac ChIP-seq datasets (including 38 primary CRC tumor samples, 9 CRC cell lines, and 4 normal colon tissues). A total of 5370 super-enhancers were identified, among which 126 super-enhancers were recurrently observed in CRC samples (**Figure 1A**). These super-enhancers were then assigned to nearby genes, and gene ontology analysis showed that these super-enhancer-regulated genes related to transcription regulation, apoptosis and cell death were identified (**Figure 1B**), which is in accordance with previous studies (6).

Next, we analyzed the gene expression profiles of these SE-related genes in CRC tumor tissues and normal colon tissues. A total of 47 genes were found to be super-enhancer regulated and malignant associated (**Figure 1C**), and these genes might be recognized as key oncogenic regulators in CRC.

### IL-20RA Is Regulated by Super-Enhancers in CRC

**Figure 2:**
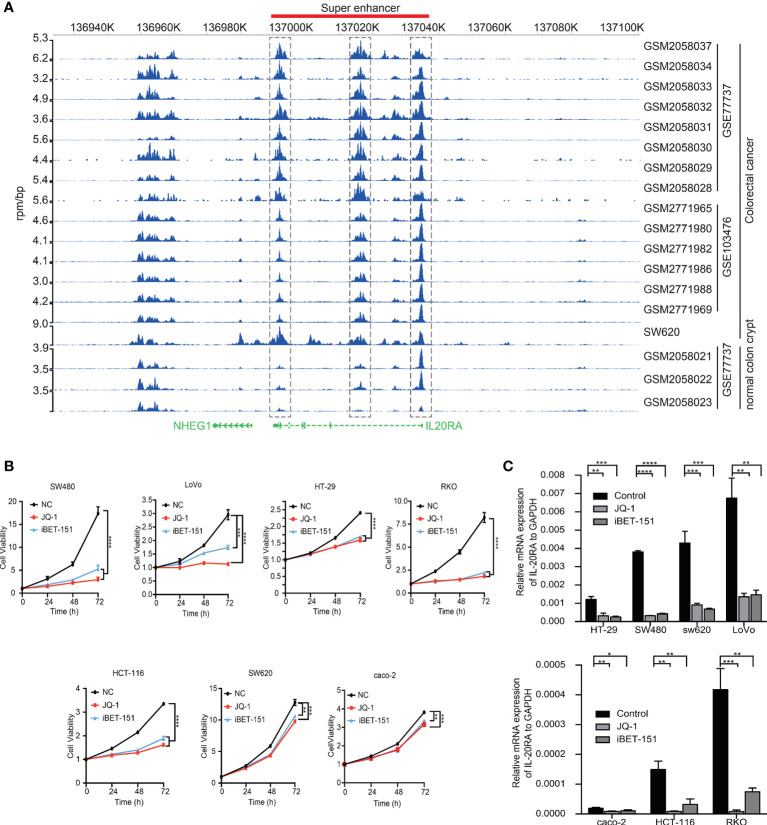
IL-20RA was activated by super-enhancers in colorectal cancer. **(A)** Genome-browser screenshot showing the binding of H3K27ac to the regulatory region of IL-20RA in primary CRC tumor cells, cell lines and normal colon tissues. **(B)** Super-enhancer inhibition decreased the cell growth of colorectal cancer cell lines. The viabilities of RKO, HT-29, and LoVo cells were detected after 0, 24, 48, and 72 hours of JQ-1 or i-BET treatment. **(C)** Super-enhancer inhibition decreased the expression of IL-20RA in CRC cells. The expression of IL-20RA was examined in RKO, HT-29, and LoVo cells after 48 hours of JQ-1 or i-BET treatment. *P < 0.05, **P < 0.01, ***P < 0.001, ****P < 0.0001.

Among the 47 oncogenic SE-regulated genes, we noticed that IL-20RA, a gene reported to regulate signal transduction was identified. Both the 5’ region, 3’ regions and intronic region of IL-20RA showed strong H3K27ac signals (**Figure 2A**). To test whether the SEs activated the expression of IL-20RA, we treated CRC cell lines (LoVo, caco-2, HT29, SW620, SW480, HCT116 and RKO) with pan-inhibitors of the SEs JQ-1 and iBET-151. We found that both inhibitors suppressed the growth of all of these cells, highlighting the essential functional super-enhancers in CRC cells (**Figure 2B**). More importantly, we found that the expression of IL-20RA was significantly downregulated after treatment with JQ-1 and iBET-151 (**Figure 2C**), indicating that the expression of IL-20RA was transcriptionally activated by SEs in CRC.

### IL-20RA Expression Is Increased in Colorectal Tumor Tissues

**Figure 3:**
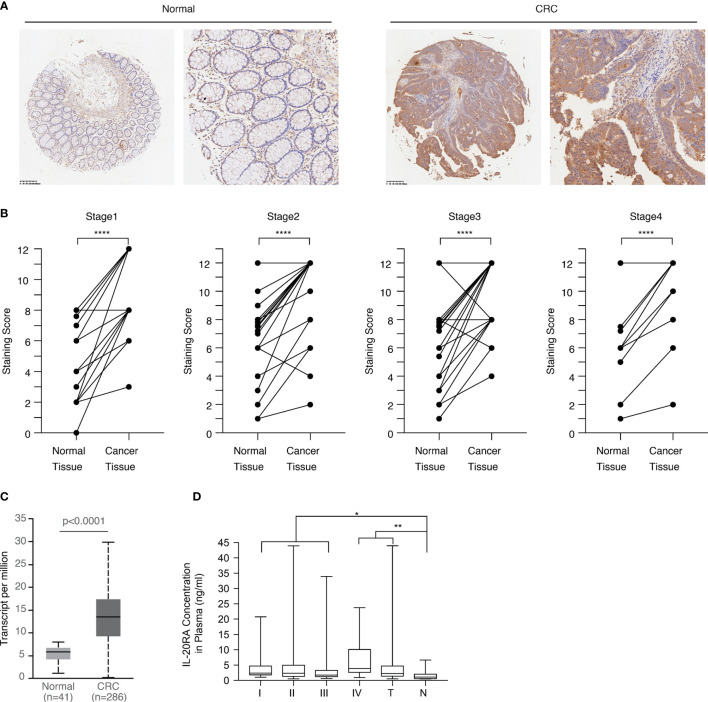
IL-20RA was highly expressed in colorectal cancer. **(A)** IHC staining images of IL-20RA between colorectal cancer tissues and paired normal tissues in the microarray. **(B)** Statistical analysis of the IHC scores of IL-20RA in colorectal cancer and paired normal tissues of 118 human colorectal cancer patients in different UICC stages. (25 for stage 1; 42 for stage 2; 40 for stage 3; 11 for stage 4). **(C)** IL-20RA was highly expressed in CRC compared to normal colon tissues. The expression of IL-20RA in CRC and normal colon tissues was retrieved from the RNA-seq data of the TCGA-COAD cohort. **(D)** IL-20RA levels in plasma between 52 colorectal cases and 17 normal persons (10 for stage 1; 16 for stage 2; 18 for stage 3; 8 for stage 4). *P < 0.05, **P < 0.01, ****P < 0.0001.

Next, we explored the clinical relevance of IL-20RA in CRC. We first analyzed the expression of IL-20RA in the tumor tissue and adjacent normal colon tissue of 118 CRC cases. The expression of IL-20RA was significantly higher in colorectal tumor tissues than in corresponding normal tissues in all stages (**Figures 3A, B**). These observations could also be validated by the TCGA-COAD cohort (**Figure 3C**), in which IL-20RA was highly expressed in tumor tissue compared to normal colon tissues. In addition, we found that higher levels of IL-20RA was positively associated with lymphatic invasion (higher 51.4% *vs.* lower 25.0%), surrounding invasion (higher 40.0% *vs.* lower 12.5%), UICC stage (higher 55.7% *vs.* lower 25.0%) and distant metastasis (higher 37.9% *vs.* lower 15.4%) (**Table 1**).

**Table 1:** Correlation between IL-20RA expression and clinical characteristics in 118 colorectal cancer tissues.

Characteristics	Cases	IL-20RA expression		P value^a^
Total cases		High	Low	
Tissue				<0.001**
Normal	118	51 (43.2%)	67 (56.8%)	
Cancer	118	101 (85.6%)	17 (14.4%)	
Age (years)				0.857
≤60	67	39 (58.2%)	28 (41.8%)	
>60	51	31 (60.8%)	20 (39.2%)	
Gender				0.575
Male	61	38 (62.3%)	23 (37.7%)	
Female	57	32 (56.1%)	25 (43.9%)	
Lymphatic invasion				0.005**
N0	70	34 (48.6%)	36 (51.4%)	
N1+N2	48	36 (75.0%)	12 (25.0%)	
Surrounding invasion				0.002**
Yes	34	28 (82.4%)	6 (17.6%)	
No	84	42 (50.0%)	42 (50.0%)	
UICC stage				0.001**
I+II	67	31 (46.3%)	36 (53.7%)	
III+IV	51	39 (76.5%)	12 (23.5%)	
Distant metastasis^b^				0.022*
Yes	28	22 (78.6%)	6 (21.4%)	
No	69	36 (52.2%)	33 (47.8%)	

a Chi-square tes.

b Including pre- and post-operation, 21 cases lost to follow-up.

*P < 0.05, **P < 0.01.

Since IL-20RA is a soluble protein and could circulate in the blood, we also collected the plasma of 52 CRC and 17 healthy donors and analyzed the plasma levels of IL-20RA in these cases. We found that the plasma levels of IL-20RA were higher in CRC than in healthy donors (**Figure 3D**). Together, these observations indicate that IL-20RA might potentially serve as a biomarker for CRC diagnosis.

### IL-20RA Is Associated to Multiple Oncogenic Pathways

**Figure 4:**
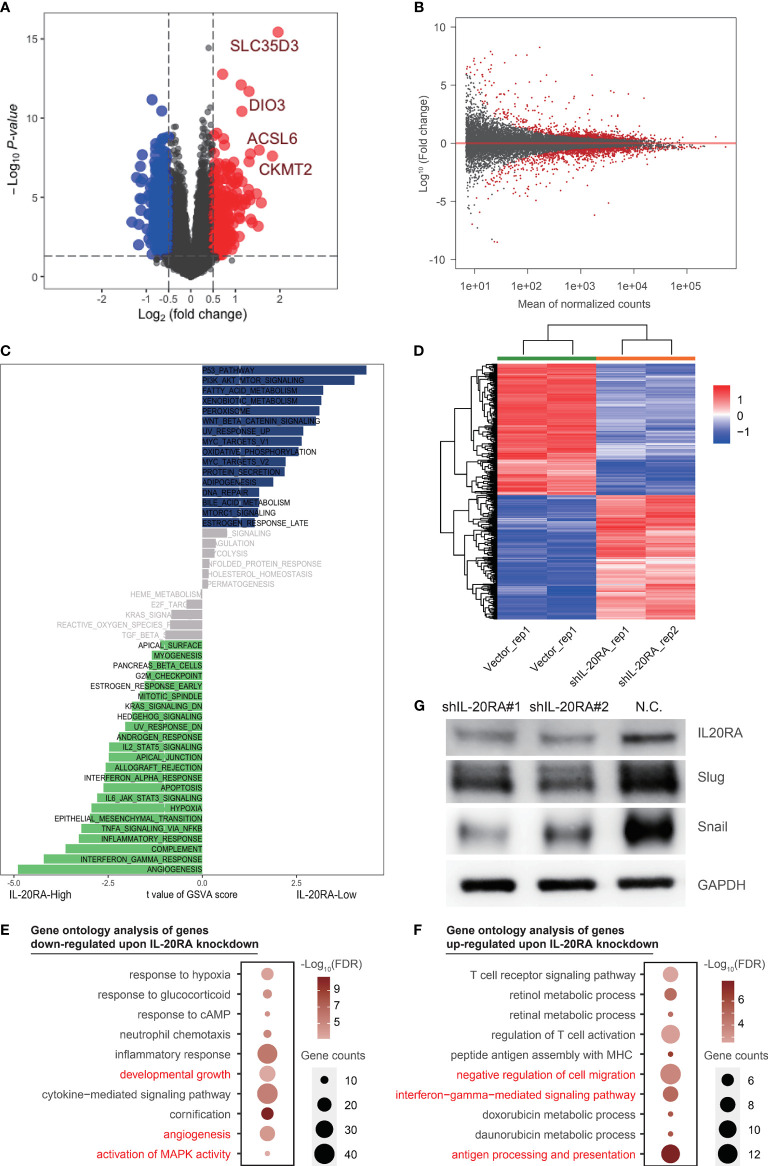
IL-20RA was associated with oncogenic and immune pathways. **(A, B)** Differently expressed genes showed by volcano plot using TCGA-COAD datasets and RNA-seq. **(C)** IL-20RA-related pathways were analyzed with Gene set variation analysis. **(D)** Heatmap showed genes differentially expressed in LoVo cells with or without IL-20RA knockdown. **(E, F)** Gene ontology analysis of genes down- **(E)** and up-regulated **(F)** post-IL-20RA knockdown. **(G)** Expression of IL-20RA, Snail and Slug at the protein level in shIL-20RA LoVo cells detected by immunoblotting. The data were obtained from 3 independent experiments and the presented as the mean ± SD (GAPDH was used as a loading control).

To further investigate and show the role of IL-20RA in CRC, we firstly explored the expression and downstream targets of IL-20RA in TCGA-COAD datasets. We found that differently expressed genes (DEGs) associated with IL-20RA, such as *SLC35D3*, *DIO3*, *ACSL6*, *CKMT2*, were concentrated on immune related genes analyzed with limma package (**Figure 4A**). Based on DEGs, Gene Ontology (GO) analysis and Gene Set Variation Analysis (GSVA) were used to investigate the related pathways and mechanisms. The results showed that IL-20RA was correlated with several classic tumor-related signaling pathways, such as KRAS, hypoxia, and EMT pathway, which might lead to promotion of tumor proliferation, migration and invasion (**Figure 4C** and **Figure S1**).

To further explore the potential function of IL-20RA in CRC, we conducted RNA-seq in LoVo cells with or without IL-20RA knockdown. A total of 642 differentially expressed genes were identified (**Figures 4B, D**). We next performed gene ontology analysis of these genes and found that genes related to developmental growth (such as *ADM*, *CSF1* and *KLF2*), angiogenesis (such as *ANGPTL4*, *FN1* and *JUN*) and activation of MAPK activity (such as *BMP2*, *DUSP6*, *MAP3K6* and *TNF*) were downregulated upon IL-20RA knockdown (**Figure 4E**), while genes related to negative regulation of cell migration (such as *COL3A1*, *FGF2*, *MCTP1* and *SLIT2*), interferon-gamma mediated signaling pathways (such as *CIITA*, *HLA*-*DPA1* and *HLA*-*DPB1*) and antigen processing and presentation (such as *HLA*-*DQB1*, *HLA*-*DRB1* and *HLA*-*DRB5*) were upregulated (**Figure 4F**), indicating that high levels of IL-20RA were related to suppressing the immune response and suggesting an essential role of IL-20RA in immune escape.

Based on bioinformatics results, we noticed that EMT pathway was positively correlated with high expression of IL-20RA, which was considered as a classic tumorigenesis signaling pathway in CRC. To confirm the downstream molecular mechanism in EMT pathway by which IL-20RA exerts its biological function, we detected the expression of Snail and Slug in LoVo cells with siIL-20RA and the negative control. The results showed that knockdown of IL-20RA in LoVo cells significantly reduced the expression of Snail and Slug, which activate EMT markers in cancer cells (**Figure 4G**).

### IL-20RA Promotes Cell Proliferation, Migration and Invasion in Colorectal Cancer

**Figure 5:**
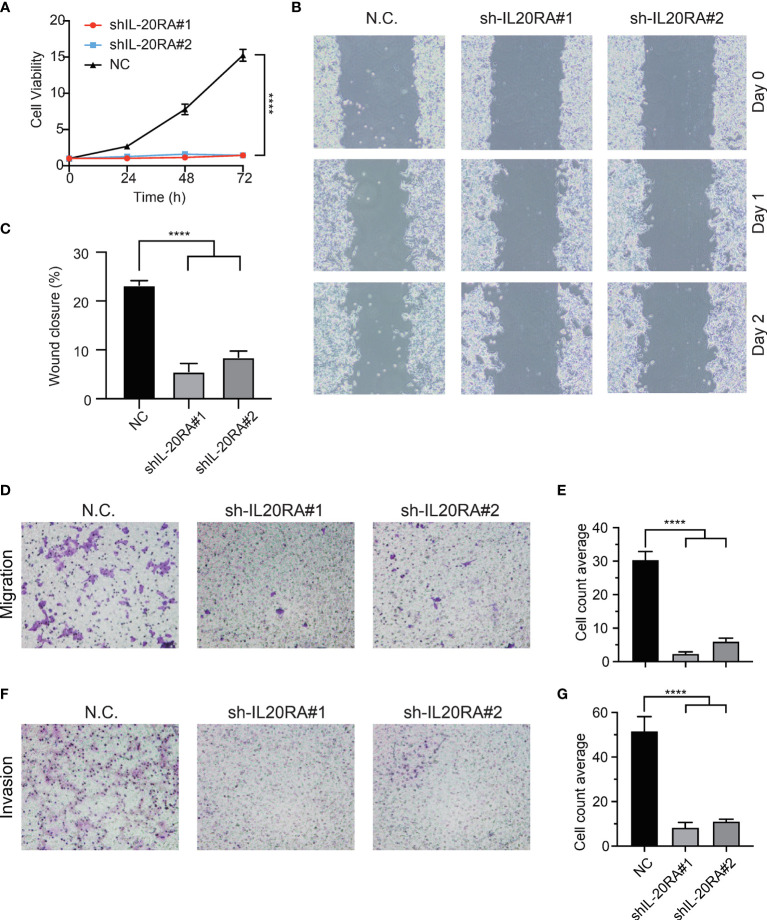
Knockdown of IL-20RA inhibited the growth and metastasis of colorectal cancer cells. **(A)** Cell proliferation of shIL-20RA was decreased in LoVo cells. Cell viability was detected at 0, 24, 48, and 72 hours by CCK-8 assay. **(B, C)** Wound healing assay of shIL-20RA in LoVo cells. Healing ability was decreased with shIL-20RA. The images were photographed at 0, 24, and 48 hours. **(D, E)** The migration ability of LoVo cells treated with shIL-20RA was decreased as shown by Transwell analyses. The number of migrated cells was counted under the average of 5 random fields. **(F, G)** The invasion ability of LoVo cells treated with shIL-20RA was decreased as shown by Transwell analyses. The number of invasive cells was counted under the average of 5 random fields. ****P < 0.0001.

To validate previous bioinformatics analysis and explore the function of IL-20RA in CRC, we knocked down the expression of IL-20RA in LoVo cells using shRNA approaches. We found that IL-20RA knockdown significantly reduced the growth of LoVo cells (**Figure 5A**). Moreover, we also found that IL-20RA knockdown suppressed the migration and invasion ability of LoVo cells (**Figures 5B–G**). Additionally, we tested whether the ligand of IL-20RA, IL-20, could affect the cell growth of CRC cells. IL-20 did not alter the growth of CRC cells, implying that IL-20RA-maintained tumor growth was exerted in a ligand-independent manner (**Figure S2**).

### IL-20RA Is Correlated With Tumor Immune Response

**Figure 6:**
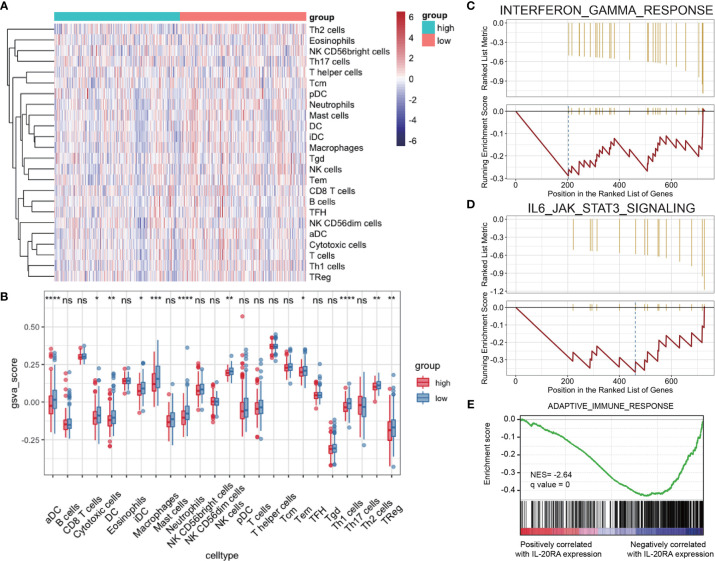
IL-20RA was related to infiltration level of immune cells. **(A, B)** Heatmap and boxplot derived from gene set variation analysis showing varied levels of infiltrating immune cells between high- and low-IL-20RA groups. **(C-E)** Gene set enrichment analysis of genes related to IFN-γ response **(C)**, IL-6-Jak-STAT3 **(D)** and adaptive immune response **(E)** in genes positively or negatively correlated with IL-20RA expression. *P < 0.05, **P < 0.01, ***P < 0.001, ****P < 0.0001, NS, no significance.

Tumor microenvironment (TME) contains multiple types of tumor-associated immune cells in solid tumors, playing important roles in tumor progression. GSVA and Gene Set Enrichment Analysis (GSEA) were used to evaluate the correlations between IL-20RA and immune cell infiltration in TME, showing that the cell infiltration level of dendritic cells, macrophages and neutrophils were negatively correlated with high expression of IL-20RA (**Figures 6A, B**). Several immune response pathways were shown to take part in the process of CRC cells regulated by IL-20RA, including interferon-gamma and IL-6-Jak-STAT3 (**Figures 6C, D**). Moreover, we found that genes related to the adaptive immune response were enriched with genes negatively correlated with IL-20RA (**Figure 6E**), suggesting that IL-20RA might suppress the immune response in CRC.

## Discussion

Our study demonstrates that SEs are highly expressed in colorectal cancer, which suggests that SEs are related to the occurrence and development of CRC. IL-20RA, driven and regulated by SEs, plays essential roles in CRC, not only in tumor progression but also prognosis. Knockdown of IL-20RA can significantly decrease the abilities of proliferation, migration and invasion *in vitro*. Genes promoting malignant phenotypes of CRC cells are decreased and correlated with knockdown of IL-20RA, while genes suppressing malignant phenotypes are increased due to RNA-seq. Moreover, IL-20RA regulates several oncogenic and immune pathways contributing to invasion and metastasis in carcinoma progression.

Enhancers are important cis-acting elements, which are small segments of DNA that can bind to proteins. They can significantly increase the frequency of gene transcription after combining with protein. With further understanding of genes, Young RA first proposed the concept of super-enhancers based on the research at that time in April 2013, which led the wave of research on SEs (5). In recent years, as research on epigenetics and tumor occurrence and development has become popular, the relationship between SEs and tumors has gradually been explored. Cancer cells can overexpress SEs through mutations, chromosomal aberrations, and high expression of oncogenic transcription factors, which in turn drive the overexpression of downstream oncogenes (24). In our study, pan-inhibitors of SEs can significantly inhibit the proliferation of multiple CRC cell lines, and we inferred that SEs mediate the progression of CRC and serve as potential anti-cancer targets. Identifying key SEs and regulated genes in CRC and looking for specifically targeting inhibitors or drugs may help suppressing tumor development, reducing side effects and providing possibilities for the treatment of CRC.

An aberrant immune response is considered to have a vital role in tumorigenesis. Knockdown of IL-20RA inhibits CRC cells proliferation, migration and invasion, implying that IL-20RA contributes to the tumorigenesis of CRC. From the bioinformatics analysis and RNA-seq, we found that genes related to oncogenic pathways and immune response were regulated by IL-20RA. For instance, angiogenesis is vital to tumor proliferation and metastasis. The tumor microenvironment stimulates tumor cells to produce proangiogenic factors and downregulate antiangiogenic factors, which breaks the balance between them (25). MAPK signaling pathways have been proven to participate in the proliferation, angiogenesis, and EMT-driven metastasis of various tumors. Besides, IFN-γ-mediated signaling pathways activate downstream signaling events, especially classical Jak-STAT signaling, inducing cell cycle arrest and apoptosis and exerting antitumor effects (26). Moreover, genes regulating antigen processing and presentation could regulate the body’s cellular and humoral immunity towards tumor cells, whose high expression enhances the immunogenicity of cancer cells and increases sensitivity towards immune checkpoint inhibitors such as anti-PD-1/anti-PD-L1 (27).

Latest research showed that IL-20RA regulated the stemness of breast cancer cells and provided immune microenvironment for the development of breast cancer. IL-20RA affected the expression of PD-L1 by activating the Jak1-STAT3-SOX2 pathway in order to reduce the immunogenicity of tumor cells and exert the function of immune evasion (11). The abnormally high expression of IL-20RA may have the similar mechanism in CRC. IL-20RA is a transmembrane protein mostly located in plasma membrane and extracellular matrix. The intracellular domain from complex combined with IL-20RB usually binds with Jak1. IL-20 family members could phosphorylate Jak1 through binding with IL-20 receptor components, and then activate STATs mediating downstream signaling pathways (28). CRC highly express IL-20RA may regulate transcription and immune response due to IL-20 family signaling. Furthermore, we can infer that IL-20RA regulates the MAPK signaling pathway based on RNA-seq. Generally, the ERK, p38, JNK, and ERK5 are major MAPK pathways involved in cellular responses through activating downstream genes (29–31). Excessive activation of JNK pathway can induce immune response of cancer cells, leading to increasing cytoskeleton regulatory proteins and regulating adhesion between focal adhesions and cell matrix (32, 33). Therefore, knockdown of IL-20RA may contribute to suppressing malignant phenotypes in CRC cells by regulating several immune signaling pathways.

Malignant solid tumors are composed of tumor cells, immune cells and stromal tissues. Immune cells in TME surrounding CRC are associated with CRC progressions and response to immune checkpoint blockage therapy, which include dendritic cells, neutrophils, macrophages, natural killer cells and so on (34, 35). Through GSVA and GSEA analysis we found that several types of immune cells including dendritic cells, macrophages, neutrophils, NK cells, Th1 cells, Th2 cells and Tregs were regulated by IL-20RA in CRC microenvironment. Tumor-associated macrophages (TAMs) and tumor-associated neutrophils (TANs) have different subgroups which exert opposite functions in tumor progressions and treatments (36). TAM has two subgroups, M1 macrophages which are anti-tumorigenic and M2 macrophages which are pro-tumorigenic. In TAN, N1 neutrophils and N2 neutrophils play opposite roles in tumorigenesis similarly as TAM. M1 macrophages are activated by several signaling pathways, such as IFN-γ and LPS, secreting inflammatory factors, chemokines and cytotoxic effectors to exert anti-tumor and immune-enhancing functions (37). N1 neutrophils secrete type I interferon and IL-18 that activates NK cells (38). In our study, TAMs, TANs and NK cells were negatively correlated with the expression of IL-20RA based on GSVA results, so we suggested that IL-20RA may decrease the infiltration of M1 macrophages and N1 neutrophils. Moreover, Th1, Th2 and Treg cells play critical roles in mediating immune response. Similarly, these cells were negatively correlated with IL-20RA expression due to GSVA results, inferring that IL-20RA may lead to immune evasion through regulating T cell recruitments.

In addition, we found that Snail and Slug, which are components of epithelial-to-mesenchymal transition (EMT), are positively correlated with IL-20RA expression. During the EMT process, epithelial tumor cells lose cell polarity, lose attachments to the basement membrane and other epithelial phenotypes, and acquire mesenchymal phenotypes, such as high migration and invasion, anti-apoptosis, and the ability to degrade extracellular matrix (39). Studies have reported that Snail and Slug can mediate cell proliferation, migration and invasion capacity in different kinds of cancer, including CRC (40, 41). Snail and Slug have been proven to be regulated by MAPK pathways especially ERK1/2 during the EMT process (40, 42). Furthermore, the Jak-STAT signaling pathway also participates in the EMT process (43, 44). Combined with the RNA-seq results, we suggest that IL-20RA regulates key transcription factors in EMT by several signaling pathways.

Our study has some potential limitations. First, we did not identify certain super-enhancers regulating the expression of IL-20RA. Specific SEs could be used for targeted therapy in CRC. Then, we could overexpress IL-20RA in CRC cell lines and investigate the malignant phenotypes compared between negative controls and overexpressed cells in order to further verify the function of IL-20RA *in vitro*. What’s more, detailed immune regulation mechanisms of IL-20RA need to be further explored. Downstream signal pathways derived from RNA-seq and bioinformatics analysis results need more experiments to be proved.

In summary, our data show that SEs drive tumorigenesis in colorectal cancer. The CRC-specific SE-driven gene IL-20RA is highly expressed in colorectal cancer tissue, leading to poor clinical characteristics and prognosis. IL-20RA knockdown could reduce cell proliferation, migration and invasion through several oncogenic and immune response pathways, suggesting that IL-20RA could be a potential target in the diagnosis and treatment of colorectal cancer.

## Data Availability Statement

The datasets presented in this study can be found in online repositories. The names of the repository/repositories and accession number(s) can be found below: Genome Sequence Archive under accession number HRA000982.

## Ethics Statement

The studies involving human participants were reviewed and approved by Human Ethics Committee of RuiJin Hospital. The patients/participants provided their written informed consent to participate in this study.

## Author Contributions

WC, RP, and MZ designed research. DY and XY performed experiments. JL and ZC performed bioinformatics analysis. CL and ZY analyzed data. DY and XY wrote the manuscript. All authors contributed to the article and approved the submitted version.

## Funding

This work was supported by the Shanghai Municipal Science and Technology Commission [grant number 19441905400] and Shanghai Jiaotong University [grant number YG2019ZDA15].

## Conflict of Interest

The authors declare that the research was conducted in the absence of any commercial or financial relationships that could be construed as a potential conflict of interest.
