# Supplementary figures and images for: Super-Enhancer Induced IL-20RA Promotes Proliferation/Metastasis and Immune Evasion in Colorectal Cancer
Source: Front Oncol. 2021 Jul 15;11:724655. doi: 10.3389/fonc.2021.724655 (PMC8319729; doi:10.3389/fonc.2021.724655)

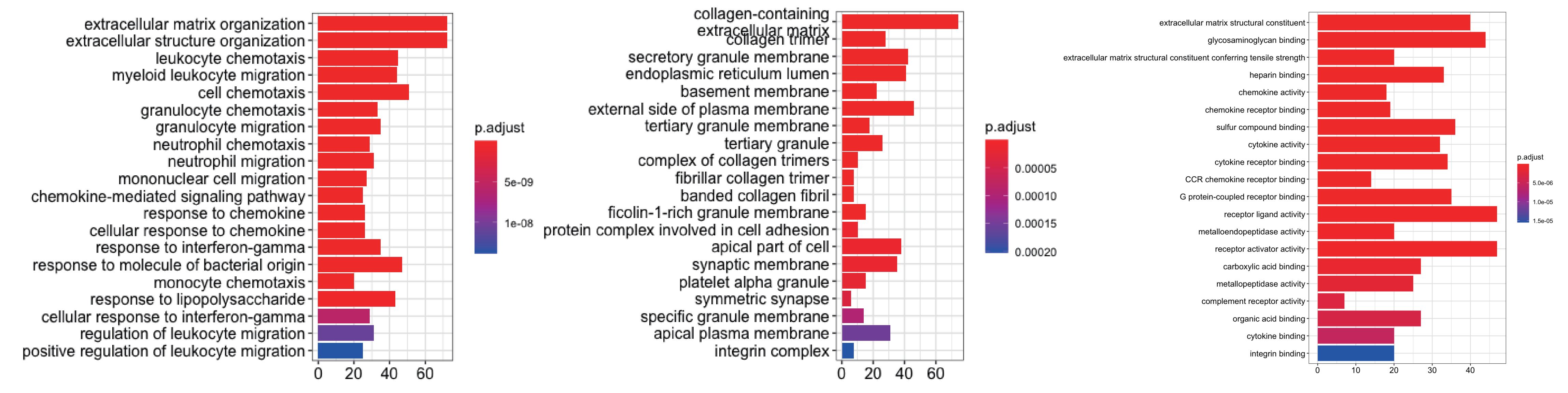

Supplement: Supplementary file 1 [file Image_1.tiff]

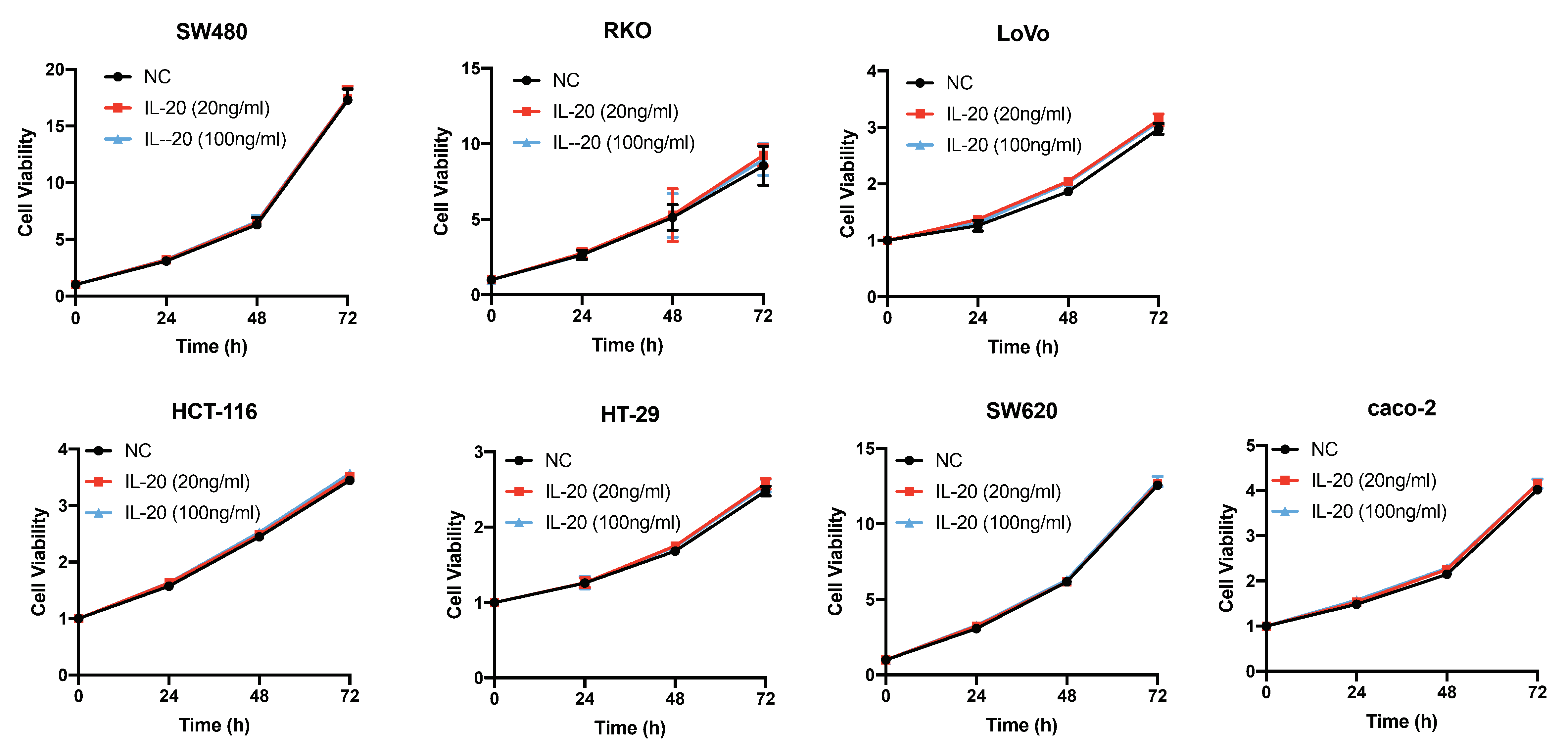

Supplement: Supplementary file 2 [file Image_2.tiff]
